# Identification of Enzalutamide-Related Genes for Prognosis and Immunotherapy in Prostate Adenocarcinoma

**DOI:** 10.1155/humu/9755727

**Published:** 2025-07-04

**Authors:** Lian Fang, Zongming Jia, Tao Zou, Ouyang Song, Jun Ouyang, Yufeng Hou, Zhiyu Zhang, Xuefeng Zhang

**Affiliations:** ^1^Department of Urology, First Affiliated Hospital of Soochow University, Suzhou, Jiangsu, China; ^2^Department of Urology, The Fourth Affiliated Hospital of Soochow University, Suzhou, Jiangsu, China

**Keywords:** biomarker, enzalutamide, gene function, machine learning, prognosis

## Abstract

Enzalutamide is classified as a novel antiandrogen medication; however, the majority of patients ultimately develop resistance to it. Consequently, conducting an in-depth investigation into potential targets of enzalutamide is essential for addressing the drug resistance observed in patients and for facilitating the discovery of new therapeutic targets. The SwissTargetPrediction database was used to identify targets linked to enzalutamide and to assess these targets in the prostate adenocarcinoma (PRAD) dataset sourced from the TCGA database. By employing various datasets and applying different machine learning methods for clustering, researchers constructed and validated both diagnostic and prognostic models for PRAD. A correlation analysis with the androgen receptor revealed TDP1 as the gene most significantly associated with enzalutamide. In addition, this study examined the relationship between TDP1 and immune infiltration. The expression levels of TDP1 and its prognostic correlation in PRAD patients were validated through immunofluorescence staining of 60 PRAD tissue specimens. Cluster analysis revealed a notable correlation among the 24 genes related to enzalutamide with regard to both prognosis and immune infiltration in PRAD patients. The diagnostic model, which incorporates various machine learning techniques, exhibits robust predictive ability for PRAD diagnosis, while the prognostic model employing the LASSO algorithm has also shown encouraging outcomes. Among the various prognostic genes linked to enzalutamide, TDP1 stands out as an important indicator of prognosis. Furthermore, immunofluorescence experiments confirmed that an increased expression of TDP1 is associated with a worse prognosis in patients with PRAD. Our results underscore the substantial potential of TDP1 as a novel diagnostic and prognostic biomarker for individuals diagnosed with PRAD.

## 1. Introduction

Prostate adenocarcinoma (PRAD) is one of the most prevalent malignancies in men, exhibiting a global rise in both diagnosis and mortality rates. According to the Global Cancer Research Agency, PRAD has overtaken lung cancer as the leading male cancer, reflecting its escalating burden [1]. Although traditional therapies such as hormone therapy, radiation, and surgical interventions have improved survival rates to a degree, their effectiveness wanes in advanced or metastatic PRAD scenarios, where the risk of recurrence remains considerable [2, 3]. This underscores the urgent need to identify innovative biomarkers and develop precision therapies to optimize early detection, personalized interventions, and prognostic assessments.

Enzalutamide, a novel androgen receptor antagonist, demonstrates promising efficacy in delaying PRAD progression, particularly for castration-resistant prostate cancer (CRPC) patients [4, 5]. Research on mechanisms shows that enzalutamide affects androgen receptor (AR) signaling via various actions. In addition to inhibiting the nuclear translocation of AR and its transcriptional activity, it competes to prevent ligand–receptor binding and interferes with AR-DNA interactions, which, in turn, reduces signals that promote tumor proliferation [6]. These properties make enzalutamide a valuable therapeutic alternative for CRPC, especially when conventional androgen deprivation fails. Clinically, enzalutamide's utility spans both standalone and combination regimens. For example, pairing it with chemotherapeutic agents may amplify tumor suppression and survival benefits [7]. Emerging evidence also highlights synergistic potential with immune checkpoint inhibitors, as enzalutamide may remodel the tumor microenvironment to potentiate immune cell activity [8]. With immunotherapy becoming a cornerstone of oncology, investigations into enzalutamide's immunomodulatory effects could unlock novel strategies to enhance treatment responses.

Integrative multiomics strategies—encompassing genomics, proteomics, transcriptomics, and metabolomics—are transforming our comprehension of the pathogenesis of PRAD. Sophisticated computational techniques facilitate the effective analysis of these complex datasets, revealing interomics relationships and providing comprehensive biological insights. Additionally, machine learning contributes to the formulation of predictive models for early diagnosis through the examination of patient omics profiles [9, 10]. This research investigated genes linked to enzalutamide in the context of PRAD diagnosis, prognosis, and treatment response. Structural data for enzalutamide were retrieved from PubChem, followed by target prediction via SwissTargetPrediction. Analysis of TCGA-PRAD data identified 24 survival-related differential genes tied to enzalutamide. Cluster analysis based on their expression patterns revealed associations with clinical outcomes, immune infiltration, and tumor staging. A diagnostic framework was established using 108 algorithmic permutations from TCGA-PRAD, validated in external cohorts. Additionally, a LASSO-optimized progression-free interval (PFI) prognostic model was developed and tested. Correlation analyses with AR highlighted TDP1 as a pivotal prognostic marker, later validated experimentally for its expression dynamics and clinical relevance in PRAD.

## 2. Materials and Methods

### 2.1. Data Sources and Sample Collection

Enzalutamide-associated targets were predicted using the SwissTargetPrediction platform. RNA sequencing data and clinical metadata from TCGA-PRAD were integrated into the analysis. Diagnostic models were developed and validated across multiple datasets, including TCGA-PRAD, GSE16120, GSE32571, GSE38241, GSE62872, and GSE6956. Sixty PRAD tissue specimens and matched adjacent noncancerous tissues were collected from Shanghai Aoduo Biotechnology Company. The tissue microarray cohort consisted of patients who underwent surgery between January 2011 and December 2014, with follow-up data collected until November 2021, covering a period of 6–10 years.

### 2.2. Nonnegative Matrix Factorization (NMF)–Based Clustering in TCGA-PRAD

The NMF algorithm uncovers biologically meaningful coefficients in gene expression matrices, grouping genes and samples to reveal latent data structures for subtype identification [11]. Subsequent differential expression analysis between Clusters A and B was implemented via the limma R package, applying thresholds of |logFC| > 0.5 and adjusted *p* < 0.05. Samples were reclustered using subcluster-derived differentially expressed genes (DEGs) via the NMF R package to explore molecular subtypes. The brunet algorithm was executed with 100 iterations per cluster number (*k* = 2–10). Optimal cluster number was selected based on cophenetic correlation, dispersion, and silhouette metrics [12].

### 2.3. Functional Profiling and Immune Infiltration Assessment

Immune infiltration levels in TCGA-PRAD samples were quantified using ssGSEA, referencing 28 immune cell-specific gene sets defined by Charoentong et al. [13]. Functional annotation was performed via Gene Ontology (GO) terms (biological processes, molecular functions, and cellular components) and KEGG pathway enrichment using the ClusterProfiler R package [14, 15]. Gene set enrichment analysis (GSEA) was additionally conducted to identify pathway-level associations [16, 17].

### 2.4. Model Development for Diagnosis and Prognosis

To develop a robust diagnostic model for PRAD, 108 machine learning algorithm combinations were tested on TCGA-PRAD training data, with performance evaluated by AUC. The top-performing model was validated in external cohorts (GSE16120, GSE32571, GSE38241, GSE62872, and GSE6956). A PFI prognostic model was constructed via LASSO regression (10-fold cross-validation) using the glmnet R package and validated in the GSE116918 dataset.

### 2.5. Immunofluorescence Staining for TDP1 Evaluation

Tissue sections were deparaffinized by immersion in xylene (two changes, 15 min each), gradually rehydrated through graded ethanol series (absolute to 75%) and distilled water. Antigen retrieval was performed in pH 9.0 EDTA buffer using a pressure cooker (2 min postboiling). After cooling, sections underwent three PBS washes (5 min each), endogenous peroxidase blocking (3% H₂O₂, 15 min), and serum-based blocking (30 min). Primary antibody incubation (TDP1, ab227144, diluted) proceeded overnight at 4°C. Following PBS washes, poly-HRP secondary antibody was applied (20 min, dark), followed by TSA fluorophore (15 min) and DAPI counterstain (10 min). Slides were imaged using fluorescence microscopy. Staining intensity (0–3) and positivity (1–4) scores were multiplied to calculate expression levels: 0–5 (*low*) and 6–12 (*high*) [18, 19].

### 2.6. Statistical Methods

TDP1 expression differences between normal and tumor tissues were assessed via Wilcoxon rank-sum test. Survival outcomes were compared using log-rank tests. Gene-stemness score correlations were analyzed using the Pearson coefficients. Statistical significance was defined as *p* < 0.05.

## 3. Result

### 3.1. Targets of Enzalutamide

**Figure 1:**
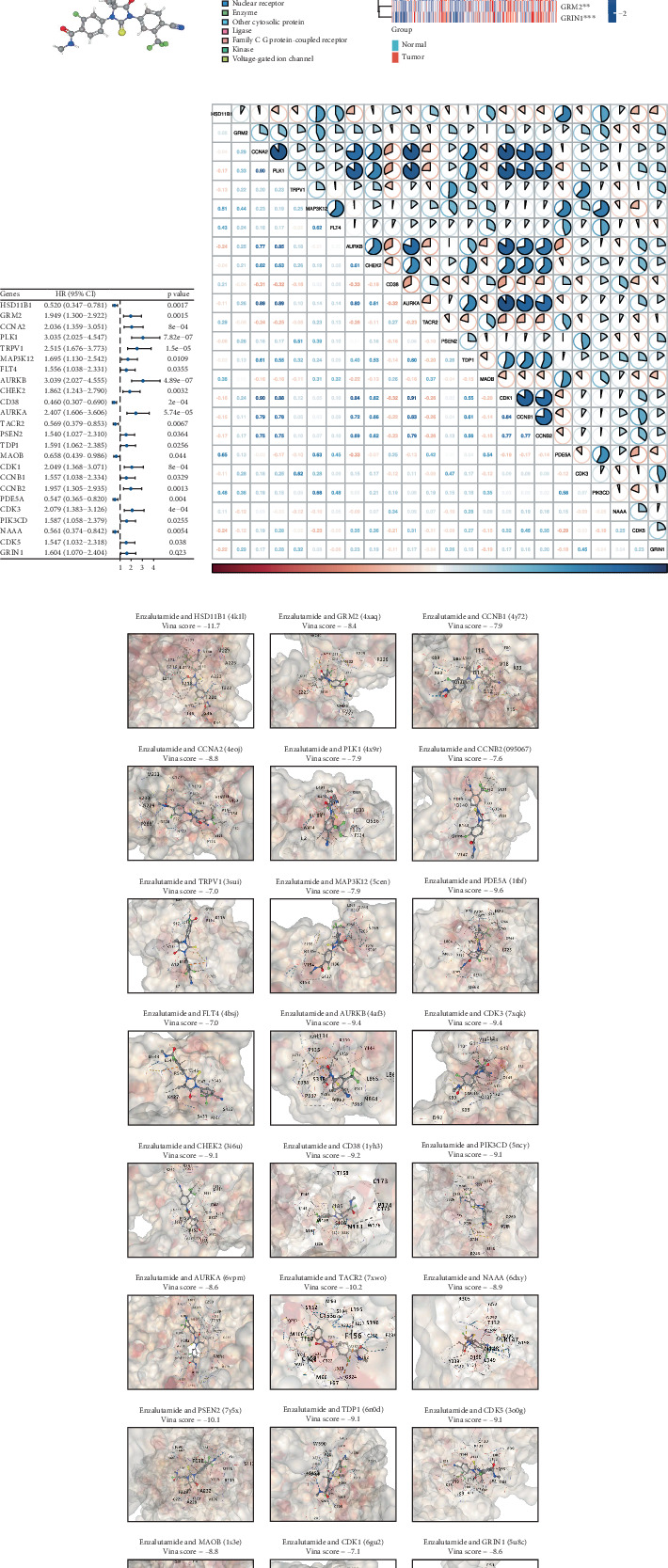
Discovery of enzalutamide-associated molecular targets in the TCGA-PRAD cohort. (a) Computational prediction of putative enzalutamide targets via SwissTargetPrediction. (b) Heatmap depicting expression patterns of enzalutamide-associated transcript profiles in tumor vs. adjacent tissues. (c) Forest plot illustrating PFI associations for enzalutamide-linked genes. (d) Correlation network analysis of enzalutamide-associated gene interactions. (e) Molecular docking validation of enzalutamide's ligand–receptor binding with candidate targets.

Enzalutamide, a second-generation antiandrogen agent, serves as a key therapeutic intervention for improving outcomes in advanced PRAD. To elucidate its molecular mechanisms, putative targets of enzalutamide were computationally predicted via the SwissTargetPrediction platform (Figure 1a), yielding 115 candidate targets. Subsequent analysis of the TCGA-PRAD cohort revealed 24 genes that showed differential expression between tumor and adjacent normal tissues and demonstrated significant associations with PFI (Figure 1b,c). Due to low mortality rates in the cohort, overall survival analysis was not prioritized. Among these 24 genes, six (HSD11B1, CD38, TACR2, MAOB, PDE5A, and NAAA) emerged as protective markers for PFI, while the other 18 correlated with adverse PFI outcomes. Correlation analysis across the TCGA-PRAD dataset demonstrated predominantly positive intergenic relationships among these targets (Figure 1d). Molecular docking simulations further validated robust interactions between enzalutamide and the 24 candidate targets, confirming their binding potential (Figure 1e).

### 3.2. Functional Analysis of Enzalutamide-Related Genes

**Figure 2:**
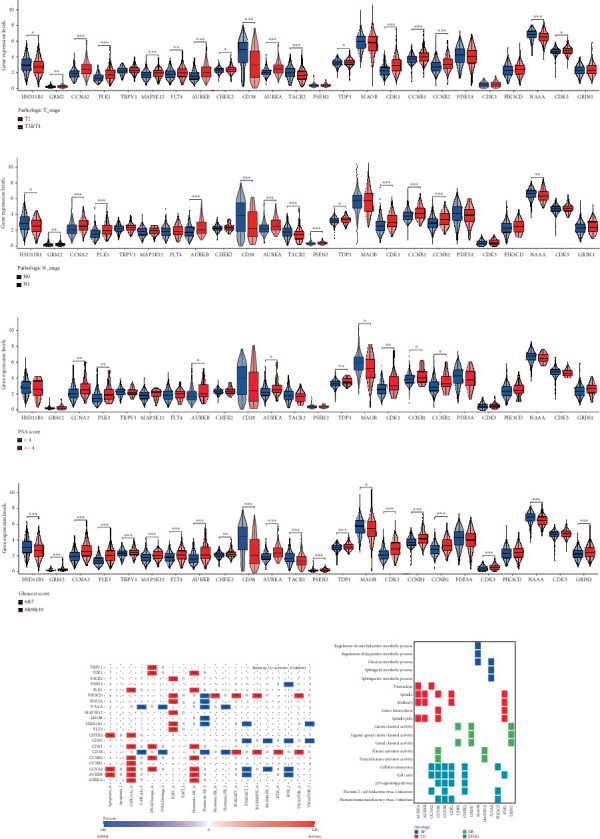
Enzalutamide-related genes play an important role in PRAD. (a) Expression analysis of enzalutamide-related genes in different T-stages. (b) Expression analysis of enzalutamide-related genes in different N-stages. (c) Expression analysis of enzalutamide-related genes in different PSA scores. (d) Expression analysis of enzalutamide-related genes in different Gleason scores. (e, f) Functional analysis of enzalutamide-related prognostic genes.

Initial investigation of the 24 genes revealed limited associations with PRAD clinicopathological parameters. Expression levels of TRPV1, PSEN2, MAOB, PDE5A, CDK3, PIK3CD, and GRIN1 showed no significant variation across T stages (Figure 2a). Similarly, TRPV1, MAP3K12, FLT4, CHEK2, MAOB, PDE5A, CDK3, PIK3CD, CDK5, and GRIN1 remained stable across N stages (Figure 2b). PSA score stratification highlighted differential expression in CCNA2, PLK1, AURKB, AURKA, TDP1, MAOB, CDK1, CCNB1, and CCNB2 (Figure 2c), while the Gleason scores showed minimal association with PDE5A, PIK3CD, or CDK5 (Figure 2d). Functional annotation via GSCA linked these genes to cell cycle regulation, DNA damage repair, and AR activation (Figure 2e). KEGG/GO analyses further confirmed cell cycle involvement (Figure 2f), with notable immune microenvironment interactions implicating B and T cell pathways.

### 3.3. Molecular Typing Based on Enzalutamide-Related Genes

**Figure 3:**
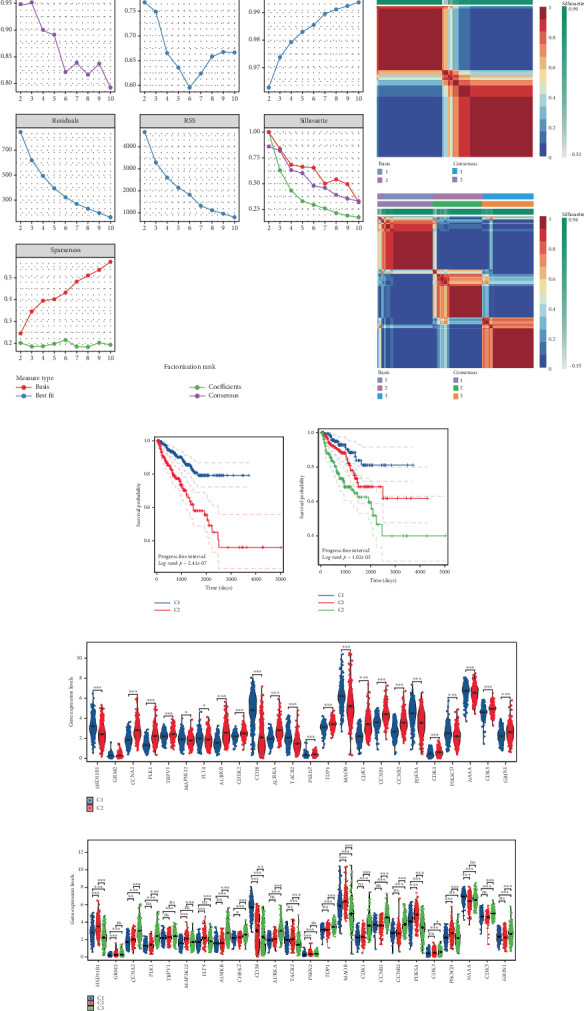
Analysis of clusters derived from genes related to enzalutamide. (a) Evaluation of both performance and stability regarding the clusters utilizing various techniques. (b) Consensus representation of NMF clustering. (c, d) Variations in survival across different clusters. (e, f) Variations in the expression levels of enzalutamide-associated genes among the distinct clusters.

To stratify TCGA-PRAD samples, we applied NMF clustering based on enzalutamide-associated gene expression. The corepresentation curve, a widely accepted metric, was used to determine optimal subgroup partitioning. The point of inflection indicating the most significant drop in this curve (noted at *k* = 5) informed the selection of clusters. However, five subgroups were deemed impractical for downstream analysis due to excessive granularity. Instead, parallel analyses were conducted using two- and three-group stratifications (Figure 3a,b). Survival analyses revealed significant PFI disparities across clusters, with maximal separation in the two-group model (Figure 3c,d). Expression profiling of the 24 enzalutamide-related genes showed minimal intercluster variability for one gene in the two-group partition versus multiple genes in the three-group system (Figure 3e,f). These findings validate the two-cluster framework as optimal for subsequent studies.

### 3.4. Immune Infiltration Correlates of Enzalutamide-Associated Genes

**Figure 4:**
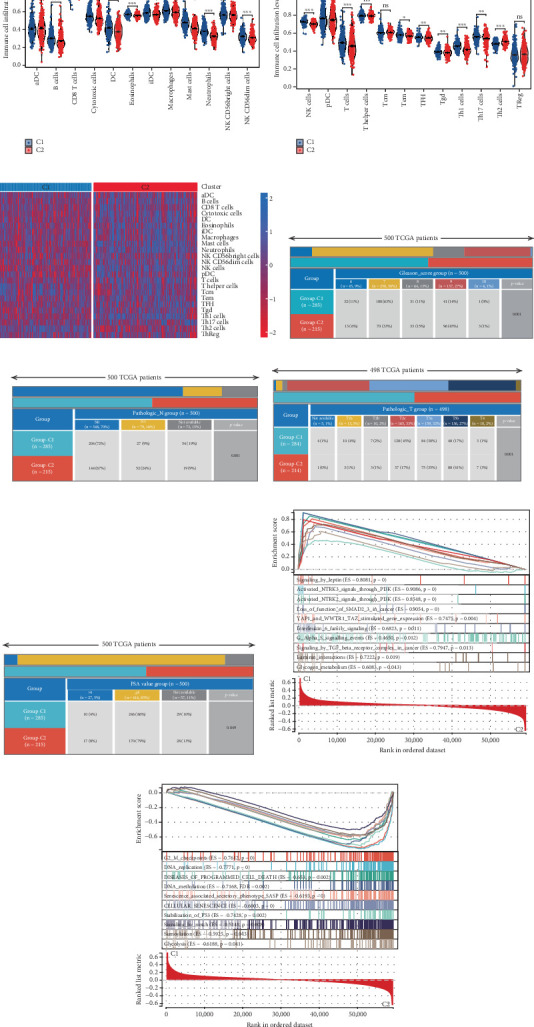
Genes associated with enzalutamide demonstrate a significant correlation with immune infiltration in PRAD. (a, b) An examination of enzalutamide-associated genes in relation to PRAD immune infiltration utilizing the ssGSEA algorithm. (c) A heatmap displaying varying levels of immune cell infiltration. (d–g) Variations in the distribution of distinct subgroups across different pathological stages of PRAD. (h, i) Gene enrichment assessment of two identified clusters.

Using signature genes for 28 immune cell populations defined by Charoentong et al., we quantified immune infiltration in TCGA-PRAD samples via the ssGSEA algorithm. Infiltration levels of aDCs, CD8+ T cells, pDCs, T helper cells, and Tcm showed no significant variation between C1 and C2 clusters. However, 19 other immune cell subsets exhibited marked infiltration disparities, indicating a robust association between enzalutamide-related genes and immune dynamics in PRAD (Figure 4a,b). Heatmap visualization further contrasted immune infiltration patterns between clusters (Figure 4c). Cluster analysis across T/N stages, Gleason scores, and PSA categories revealed distinct clinical subgroup distributions (Figures 4d, 4e, 4f, 4g). Functional enrichment analysis linked C1 to SMAD2 pathway suppression and C2 to G2M checkpoint activation (Figure 4h,i).

### 3.5. Development of an Enzalutamide-Associated Diagnostic Model

**Figure 5:**
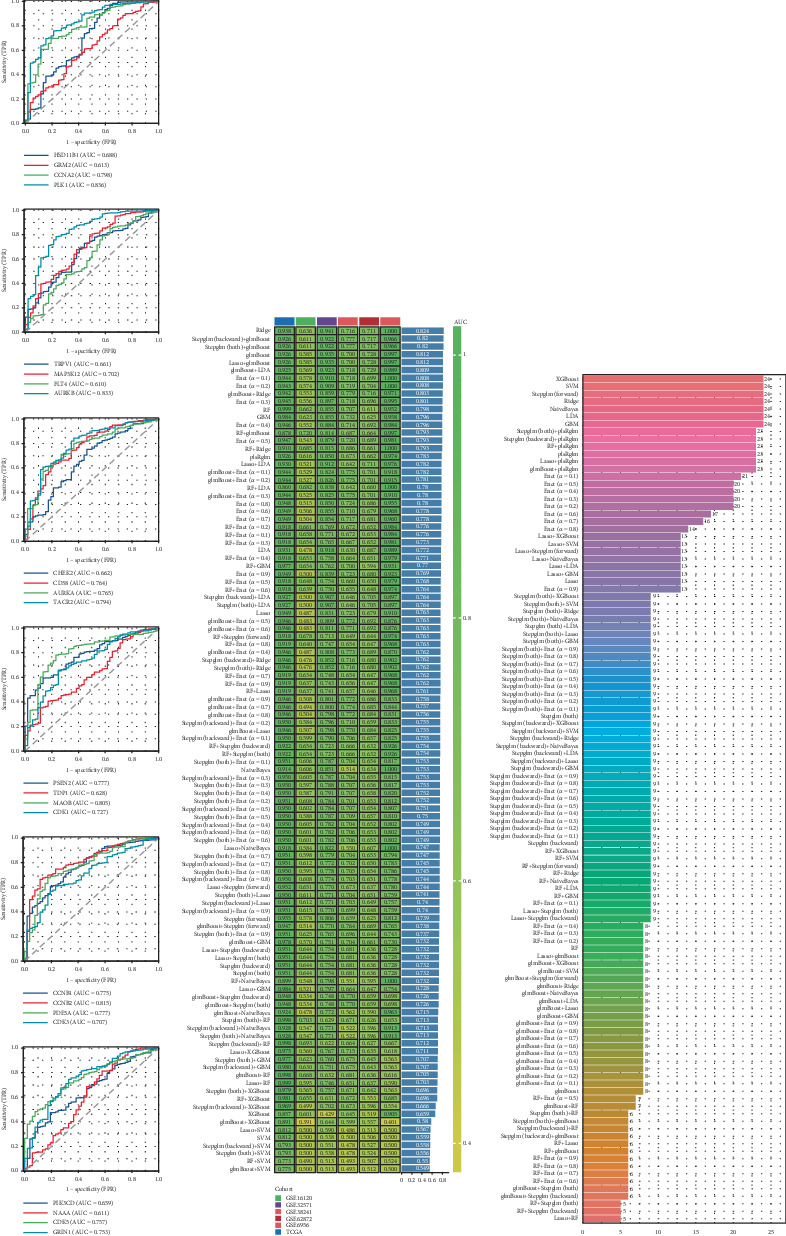
The combination of the Ridge algorithm is regarded as the optimal choice for developing diagnostic models. (a) An analysis of the predictive capabilities of enzalutamide-related genes in the diagnosis of PRAD patients. (b) AUC scores for diagnostic models generated through different algorithm combinations. (c) The number of genes incorporated into diagnostic models produced with various algorithm combinations.

To explore the clinical utility of enzalutamide-related genes in PRAD, we assessed their diagnostic potential using receiver operating characteristic (ROC) curve analysis. These genes demonstrated robust discriminative power for PRAD detection (Figure 5a). Leveraging six PRAD datasets (TCGA-PRAD for training; GSE16120, GSE32571, GSE38241, GSE62872, and GSE6956 for validation), we developed a diagnostic framework. Among 108 algorithm permutations tested, Ridge regression achieved optimal performance, yielding a training set AUC of 0.938. Validation cohort AUCs were 0.636 (GSE16120), 0.941 (GSE32571), 0.716 (GSE38241), 0.711 (GSE62872), and 1.0 (GSE6956) (Figure 5b). Notably, all 24 enzalutamide-linked genes were incorporated into the final Ridge-derived model (Figure 5c).

### 3.6. LASSO-Derived Prognostic Framework

**Figure 6:**
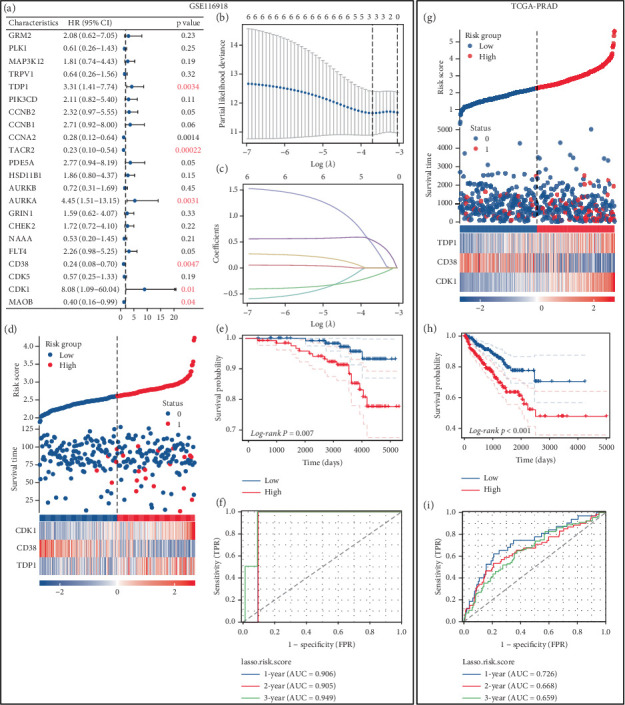
Development of a prognostic model. (a) A forest plot depicting the prognosis of PFI concerning genes linked to enzalutamide. (b, c) Development of prognostic models employing the LASSO algorithm. (d) A heatmap representing the expression levels of genes included in the prognostic model sourced from the GSE116918 dataset. (e) Differences in survival rates identified between high-risk and low-risk patient cohorts within the GSE116918 dataset. (f) The importance of the risk score from the GSE116918 dataset in predicting prognosis for individuals diagnosed with PRAD. (g) A heatmap that reveals the expression levels of genes constituting the prognostic model from the TCGA-PRAD dataset. (h) Prognostic variations recorded between high-risk and low-risk groups in the TCGA-PRAD dataset. (i) The relevance of the risk score from the TCGA-PRAD dataset in evaluating prognosis for patients with PRAD.

To explore the prognostic relevance of enzalutamide-associated genes in PRAD, we integrated the GSE116918 cohort, which includes metastasis-linked clinical data. This cohort profiles 22 enzalutamide-related transcripts. Survival analysis with metastasis as the endpoint identified six PFI-associated genes (Figure 6a). LASSO regression refined these to a three-gene signature (CDK1, CD38, and TDP1), demonstrating robust prognostic performance (Figure 6b,c). Expression heatmaps, Kaplan–Meier survival curves, and ROC analyses stratified patients into high- and low-risk groups, revealing significantly shorter PFI in high-risk individuals (Figures 6d, 6e, 6f). Subsequent validation in TCGA-PRAD confirmed poorer PFI outcomes for high-risk patients, validating its clinical utility (Figures 6g, 6h, 6i).

### 3.7. Immune Infiltration Patterns Linked to Prognostic Risk Stratification

**Figure 7:**
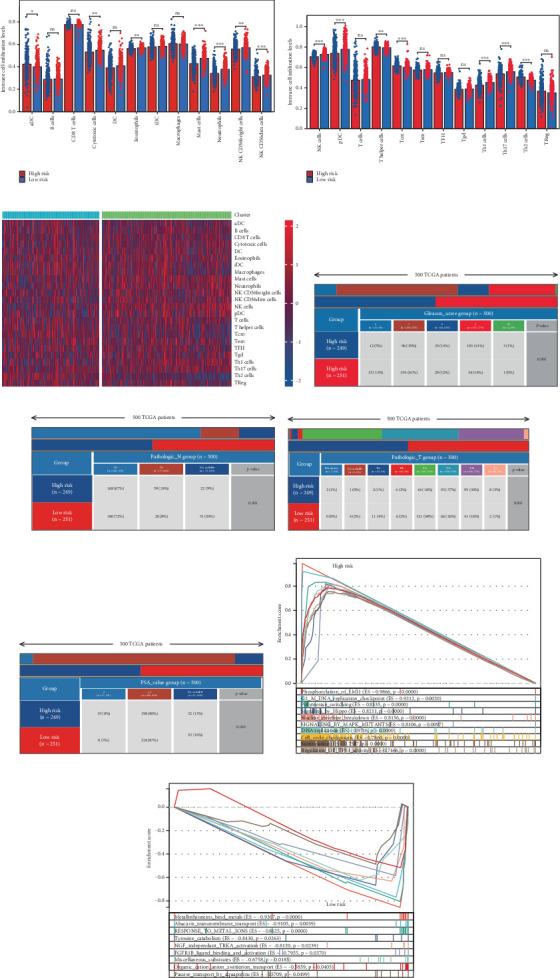
The prognostic risk score shows a significant correlation with immune infiltration in PRAD. (a, b) An analysis of the prognostic risk score concerning immune infiltration in PRAD was conducted using the ssGSEA methodology. (c) A heatmap displays diverse levels of immune cell infiltration. (d–g) Variations in the distribution of various subgroups across distinct pathological stages of PRAD are presented. (h, i) Gene enrichment analysis was performed on two different groups.

Using the ssGSEA algorithm, immune infiltration levels across cell subtypes in TCGA-PRAD were evaluated. B cells, CD8+ T cells, dendritic cells (DC), immature DCs (iDC), macrophages, T cells, memory T cells (Tem), T follicular helper cells (TFH), gamma delta T cells (Tgd), and regulatory T cells (TReg) showed no significant disparities between high- and low-risk groups. Conversely, 14 other immune cell subsets exhibited marked infiltration differences, underscoring distinct relationships between the enzalutamide-based prognostic model and PRAD immune dynamics (Figure 7a,b). Heatmaps further contrasted immune cell abundance between risk categories (Figure 7c). Clinical parameter distributions (T/N stages, Gleason scores, and PSA clusters) aligned with risk stratification (Figures 7d, 7e, 7f, 7g). Enrichment analyses revealed high-risk group association with EMI1 phosphorylation and low-risk linkage to metal-binding metallothioneins (Figure 7h,i).

### 3.8. TDP1 Identified as the Most Important Gene in Enzalutamide-Related Genes

**Figure 8:**
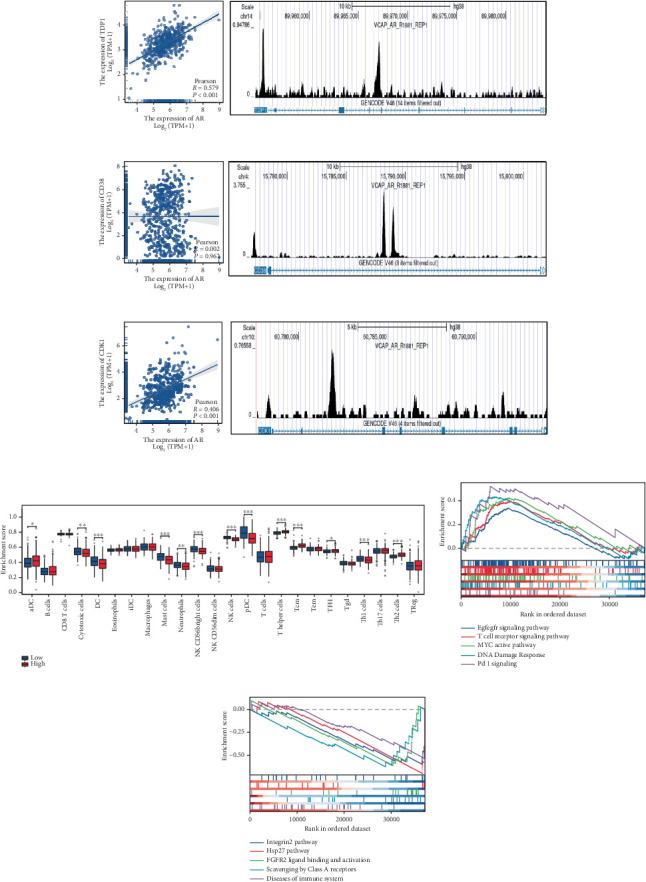
TDP1 is the most essential gene among enzalutamide-related genes. (a–c) The correlation between enzalutamide-related genes and AR was analyzed. (d) An examination of the relationship between TDP1 and immune infiltration in PRAD. (e–f) Evaluative analysis of TDP1's functions.

Clinical evidence confirms that endocrine therapies for PRAD primarily focus on androgen axis disruption, where reactivated AR signaling drives therapeutic resistance and tumor advancement. In our prognostic framework, we evaluated associations between three prognostic model genes (CDK1, CD38, and TDP1) and AR activity. TDP1 and CDK1 showed positive AR correlations (Pearson *r* = 0.579 for TDP1), contrasting with CD38's nonsignificant association (Figures 8a, 8b, 8c). AR binding site analysis revealed significant promoter region enrichment for TDP1 and CDK1, particularly at TDP1 loci, aligning with its transcriptional regulatory role. These results nominate TDP1 as the central enzalutamide-associated hub gene and a potential therapeutic target in PRAD. Immune profiling linked elevated TDP1 expression to altered infiltration patterns across multiple immune subsets (Figure 8d). Pathway enrichment further demonstrated that TDP1-high tumors showed activation of PD-1, EGFR, and MYC pathways alongside suppression of integrin *β*2 and HSP27 signaling (Figure 8e,f).

### 3.9. Validation of TDP1 Expression and Prognostic Differences

**Figure 9:**
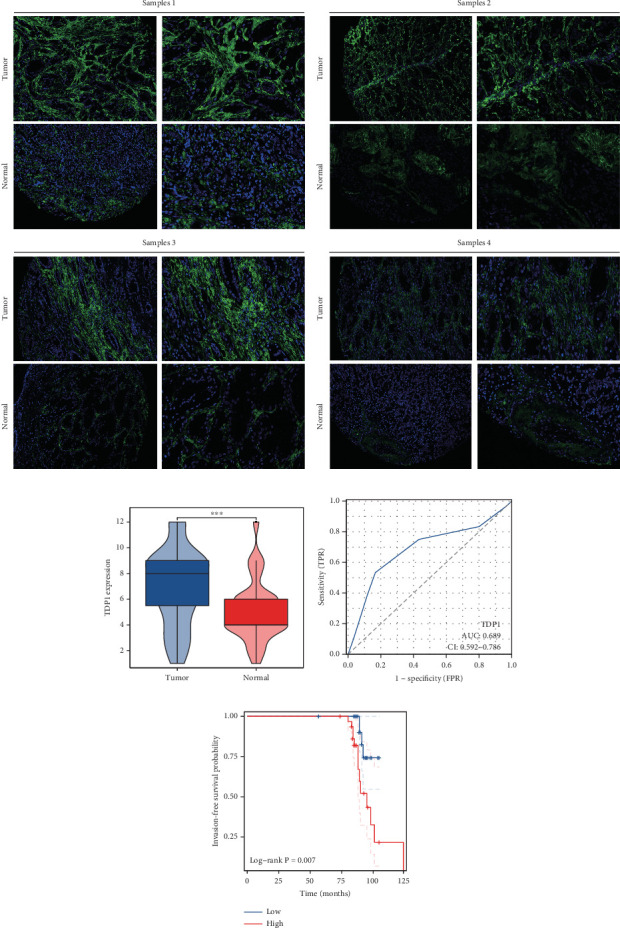
TDP1 is highly expressed in PRAD. (a, b) Variations in TDP1 expression within PRAD. (c) ROC curve analysis indicating the diagnostic predictive value of TDP1. (d) Survival analysis KM curve reflecting invasion-free survival associated with TDP1 in PRAD.

Our study underscores the clinical significance of TDP1, a gene implicated in enzalutamide response, through multimodal validation. A cohort of 60 PRAD specimens paired with adjacent normal tissues was analyzed. Immunofluorescence staining (nuclear counterstain: blue; TDP1: green) revealed pronounced TDP1 upregulation in tumors versus benign tissues (Figure 9a). Boxplot visualization further quantified this differential expression (Figure 9b). ROC analysis demonstrated strong diagnostic utility for TDP1 (AUC = 0.689, Figure 9c), while survival modeling linked elevated TDP1 levels to reduced invasion-free intervals in patients with vascular involvement (log-rank *p* < 0.01, Figure 9d).

## 4. Discussion

PRAD, a clinically aggressive malignancy, is frequently diagnosed at advanced stages with metastatic spread, contributing to its unfavorable prognosis [20]. This clinical reality emphasizes the urgent need for biomarkers enabling early detection and therapeutic monitoring, which are fundamental to advancing precision oncology strategies [21]. While enzalutamide—a second-generation AR antagonist—has emerged as a therapeutic breakthrough for CRPC, its efficacy is limited by the emergence of resistance mechanisms such as AR mutations, compensatory pathway activation, and genomic adaptations [22]. Our study explores enzalutamide-associated molecular targets to identify novel biomarkers for improving diagnostic accuracy, prognostic stratification, and immunotherapeutic approaches in PRAD.

Using the SwissTargetPrediction platform, we identified 115 genes with potential enzalutamide-binding capacity, with AR exhibiting the highest predicted binding affinity [23, 24]. This aligns with enzalutamide's dual mechanism, competitively inhibiting androgen–AR binding while promoting receptor degradation, thereby reducing AR-driven oncogenic signaling [25]. Analysis of TCGA-PRAD data revealed 24 DEGs with prognostic significance. Key candidates include AURKB, whose suppression impedes PRAD cell proliferation via apoptosis induction [26]. CD38, linked to growth arrest through apoptotic pathways, and AURKA are targeted by alisertib to inhibit AR-mediated tumor growth [27]. TACR2 modulates Wnt/*β*-catenin signaling to restrict migration and proliferation [28].

Molecular docking via CB-Dock2 confirmed strong enzalutamide binding for all 24 candidates (Vina scores < −7.0, a threshold indicating high-affinity interactions) [29]. Functional enrichment highlighted associations with cellular senescence—a stress adaptation mechanism characterized by cell cycle arrest, metabolic reprogramming, and senescence-associated secretory phenotypes (SASP) [30]. Senescent cells frequently demonstrate resistance to therapy due to improved DNA repair processes and heightened antioxidant defenses, which may clarify the mechanisms behind enzalutamide resistance [31]. These findings validate the biological relevance of our identified gene set in PRAD pathogenesis.

Machine learning approaches, particularly non-NMF, stratified TCGA-PRAD patients into two molecular subgroups with distinct PFI outcomes [32]. Cluster 2, enriched for p53 instability and senescence pathways, correlated with poorer prognosis. Diagnostic and prognostic models built using Ridge and LASSO algorithms demonstrated robust performance across validation cohorts (AUC: 0.636–1.0; log-rank *p* < 0.01), underscoring their clinical potential.

Notably, TDP1 emerged as the most AR-correlated gene (*r* = 0.579), with AR binding site enrichment in its promoter region. Immunofluorescence validation in 60 PRAD specimens confirmed TDP1 overexpression in tumors versus normal tissues (AUC = 0.689) and its association with reduced invasion-free survival (*p* < 0.01). Pathway analyses linked high TDP1 to PD-1/EGFR/MYC activation and integrin *β*2/HSP27 suppression, positioning it as a promising therapeutic target. While our multiomics approach provides novel insights, the findings are primarily derived from TCGA and GEO datasets. Future validation in larger and more diverse cohorts, as well as functional studies, is necessary to confirm their translational relevance.

## 5. Conclusion

This research represents the initial exploration of enzalutamide-associated targets concerning the diagnosis, prognosis, and immune infiltration in PRAD. Notably, TDP1 stands out as the most crucial gene and may serve as a new diagnostic and prognostic marker for individuals diagnosed with PRAD.

## Data Availability

The data that support the findings of this study are available from the corresponding authors upon reasonable request.
